# Preoperative and Intraoperative Predictors of Direct Home Discharge in Patients with Acute Type A Aortic Dissection: A Single-Center Retrospective Observational Study

**DOI:** 10.5761/atcs.oa.26-00043

**Published:** 2026-08-27

**Authors:** Yoshifumi Kono, Yukio Mikami, Naoki Mio, Kana Kanai, Shigehito Shiota, Seimei Kure, Taiichi Takasaki, Shinya Takahashi

**Affiliations:** 1 Division of Rehabilitation, Department of Clinical Practice and Support, Hiroshima University Hospital, Hiroshima, Hiroshima, Japan; 2 Department of Rehabilitation Medicine, Hiroshima University Hospital, Hiroshima, Hiroshima, Japan; 3 Department of Cardiovascular Surgery, Hiroshima University Hospital, Hiroshima, Hiroshima, Japan; 4 Department of Surgery, Graduate School of Biomedical and Health Sciences, Hiroshima University, Hiroshima, Hiroshima, Japan

**Keywords:** aortic dissection, patient discharge, risk factors

## Abstract

**Purpose:**

This study aimed to identify the preoperative and intraoperative predictors of direct home discharge in patients following surgical repair for acute type A aortic dissection (ATAAD).

**Methods:**

We included patients with ATAAD who underwent surgical repair between 2012 and 2025. Patients were categorized into 2 groups based on their discharge destination. Multivariable logistic regression analysis was performed to identify independent predictors of direct home discharge.

**Results:**

Among 277 patients, 133 (48.01%) were discharged directly home. Multivariable logistic regression analysis revealed that age (per year, odds ratio [OR], 0.961; 95% confidence interval [CI], 0.936–0.986; P = 0.003), living alone (OR, 0.523; 95% CI, 0.277–0.985; P = 0.045), cardiac tamponade (OR, 0.434; 95% CI, 0.203–0.927; P = 0.031), and neurological deficits of the extremities (OR, 0.369; 95% CI, 0.161–0.845; P = 0.018) were significant independent predictors of direct home discharge. The area under the receiver operating characteristic curve was 0.731.

**Conclusion:**

Advanced age, living alone, preoperative cardiac tamponade, and neurological deficits of the extremities are significant predictors of a reduced likelihood of direct home discharge following surgical repair for ATAAD.

## Introduction

Acute type A aortic dissection (ATAAD) remains a life-threatening cardiovascular event with high mortality despite recent advances in diagnosis and management.^1)^ Although surgical repair has significantly improved survival,^1,2)^ survivors often experience prolonged hospitalization and difficulties in returning directly home. Indeed, a nationwide study reported that up to one-third of these patients are discharged to non-home destinations.^3)^

Historically, clinical evaluations of ATAAD outcomes have focused predominantly on mortality and major morbidity, often at the expense of functional recovery. While home discharge from the primary hospital serves as a key indicator of successful functional restoration and reduced readmission risk,^4,5)^ its predictors remain underexplored. Identifying these predictors is essential for initiating proactive, personalized discharge coordination at the earliest possible stage—specifically, before postoperative complications manifest.

Thus, this study aimed to identify the preoperative and intraoperative predictors of direct home discharge in patients following surgical repair for ATAAD, providing clinicians with a basis for early risk stratification and resource allocation.

## Materials and Methods

### Study design

This single-center retrospective observational study included patients with ATAAD who underwent surgical repair at our hospital between January 2012 and December 2025. The key exclusion criteria were pre-event activities of daily living (ADLs) dependency and incomplete data. Eligible patients were categorized based on their discharge destination into 2 groups: direct home discharge and non-home discharge (comprising transfers to post-acute care facilities and in-hospital deaths). Discharge destination was determined by multidisciplinary consensus (including surgeons, nurses, and rehabilitation specialists) based on medical stability and functional independence relative to the home environment. This study was conducted in accordance with the principles of the Declaration of Helsinki and was approved by the Ethical Committee for Epidemiology of Hiroshima University (approval number: E2021-2790). The requirement for written informed consent was waived, and an opt-out method was employed.

### Data collection

Data, including demographic characteristics, medical history, preoperative status, surgical information, and postoperative outcomes, were collected from medical records. Preoperative variables consisted of clinical manifestations—including cardiac tamponade, cardiac arrest, severe aortic valve regurgitation, coma, and neurological deficits of the extremities—and baseline laboratory data, including hemoglobin, serum creatinine, and serum albumin. Cardiac tamponade was defined as cardiogenic shock with pericardial effusion and a systolic blood pressure ≤90 mmHg. Intraoperative data, including aortic repair strategies, concomitant procedures, operation time, cardiopulmonary bypass time, aortic cross-clamp time, and intraoperative fluid balance, were obtained. Concomitant procedures included the Bentall procedure, coronary artery bypass grafting, aortic valve replacement, and other procedures. Postoperative outcomes included the length of hospital stay and the Barthel Index (BI) at discharge, which evaluates independence in ADLs.^6)^

### Statistical analyses

JMP Pro version 18.0.1 (SAS Institute Inc., Cary, NC, USA) was used for all statistical analyses. Continuous variables were compared using the unpaired Student’s t-test or the Mann–Whitney U test, depending on the normality of the distribution. Categorical variables were compared using the chi-square test or Fisher’s exact test, as appropriate. Subsequently, variables with a P-value <0.10 in the univariable analyses were included in a multivariable logistic regression analysis to identify independent predictors of direct home discharge. To avoid multicollinearity, operation time, cardiopulmonary bypass time, and aortic cross-clamp time were not included in the same model. Cardiopulmonary bypass time was prioritized based on its superior clinical relevance and established prognostic value in cardiac surgery outcomes.^7–9)^ Results were presented as odds ratios (ORs) with 95% confidence intervals (CIs). A P-value <0.05 was considered statistically significant.

## Results

**Fig. 1:**
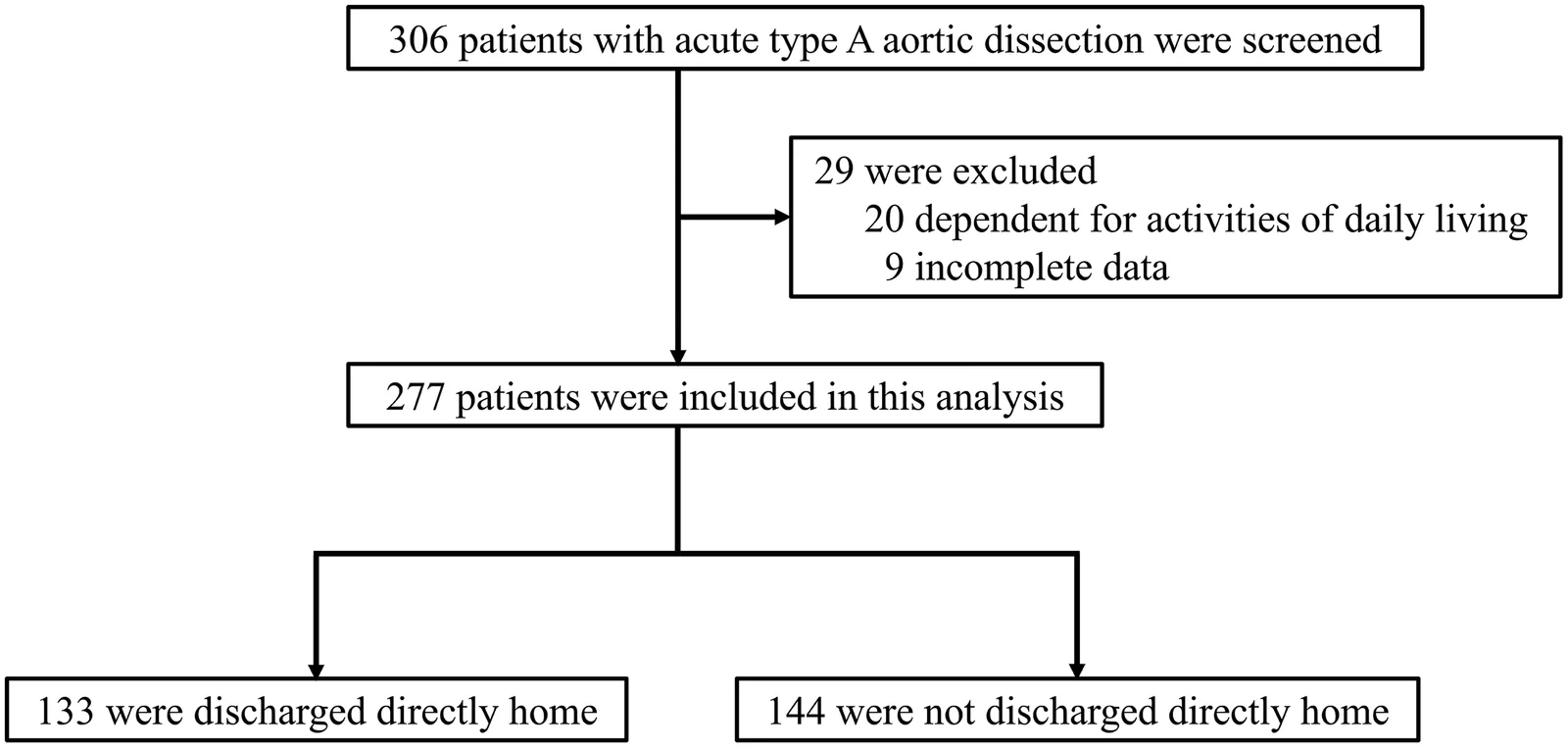
Flowchart of patient selection

A total of 306 patients were screened during the study period. Of these, 29 were excluded due to pre-event ADL dependency (n = 20) or incomplete data (n = 9). Of the 277 patients included in this study, 133 (48.01%) were discharged directly home (**Fig. 1**).

Significant differences were observed between the direct home discharge and non-home discharge groups across several domains: preoperatively in age (P <0.001), living alone (P = 0.025), cardiac tamponade (P <0.001), coma (P = 0.004), neurological deficits of the extremities (P = 0.006), serum creatinine (P = 0.019), and serum albumin (P = 0.005); intraoperatively in concomitant procedures (P = 0.049), cardiopulmonary bypass time (P = 0.049), and fluid balance (P = 0.009); and postoperatively in BI at discharge (P <0.001) (**Table 1**).

**Table 1:** Patient demographics and perioperative data

	Direct home discharge (n = 133)	Non-home discharge (n = 144)	P-value
Demographic characteristics and history			
Age (years)	65.16 ± 12.79	71.08 ± 12.21	<0.001
Male	73 (54.89%)	73 (50.69%)	0.485
Body Mass Index (kg/m^2^)	23.78 ± 4.05	23.59 ± 4.10	0.646
Diabetes mellitus	6 (4.51%)	10 (6.94%)	0.386
Chronic kidney disease	8 (6.02%)	12 (8.33%)	0.456
Hemodialysis	3 (2.26%)	3 (2.08%)	>0.999
COPD	4 (3.01%)	10 (6.94%)	0.173
Previous cardiac surgery	8 (6.02%)	11 (7.64%)	0.593
Living alone	22 (16.54%)	40 (27.78%)	0.025
Preoperative data			
Cardiac tamponade	14 (10.53%)	38 (26.39%)	<0.001
Cardiac arrest	1 (0.75%)	7 (4.86%)	0.068
Severe aortic valve regurgitation	7 (5.26%)	10 (6.94%)	0.560
Coma	2 (1.50%)	14 (9.72%)	0.004
Neurological deficits of the extremities	12 (9.02%)	30 (20.83%)	0.006
Hemoglobin (g/dL)	12.64 ± 1.95	12.21 ± 1.93	0.061
Serum creatinine (mg/dL)	1.06 ± 1.12	1.22 ± 1.11	0.019
Serum albumin (g/dL)	3.66 ± 0.50	3.52 ± 0.47	0.005
Intraoperative data			
Ascending aortic/Hemiarch replacement	45 (33.83%)	38 (26.39%)	0.177
Total arch replacement	88 (66.17%)	106 (73.61%)	
Concomitant procedures	16 (12.03%)	30 (20.83%)	0.049
Operation time (min)	362.35 ± 98.21	390.27 ± 123.19	0.083
Cardiopulmonary bypass time (min)	197.65 ± 57.55	222.46 ± 90.57	0.049
Aortic cross-clamp time (min)	108.89 ± 35.44	118.79 ± 47.09	0.154
Intraoperative fluid balance (mL)	3837.07 ± 1952.32	4633.26 ± 2506.31	0.009
Postoperative outcomes			
Hospital stay (days)	25 (20–33)	28 (22–40.25)	0.066
Barthel Index at discharge (points)	100 (100–100)	45 (0–76.25)	<0.001

Values are mean ± SD, n (%), or median (IQR).

COPD, chronic obstructive pulmonary disease; SD, standard deviation; IQR, interquartile range

Multivariable logistic regression analysis revealed that age (per year, OR, 0.961; 95% CI, 0.936–0.986; P = 0.003), living alone (OR, 0.523; 95% CI, 0.277–0.985; P = 0.045), cardiac tamponade (OR, 0.434; 95% CI, 0.203–0.927; P = 0.031), and neurological deficits of the extremities (OR, 0.369; 95% CI, 0.161–0.845; P = 0.018) were significant independent predictors of direct home discharge (**Table 2**). The area under the receiver operating characteristic curve (AUC) was 0.731.

**Table 2:** Logistic regression analysis of direct home discharge

	Univariable			Multivariable		
	OR	95% CI	P-value	OR	95% CI	P-value
Age (per 1 year)	0.963	0.943–0.982	<0.001	0.961	0.936–0.986	0.003
Male	1.183	0.738–1.901	0.485			
Body Mass Index (per 1 kg/m^2^)	1.012	0.955–1.073	0.688			
Diabetes mellitus	0.633	0.210–1.756	0.383			
Chronic kidney disease	0.704	0.268–1.759	0.455			
Hemodialysis	1.085	0.198–5.950	0.922			
COPD	0.416	0.112–1.276	0.128			
Previous cardiac surgery	0.774	0.291–1.974	0.592			
Living alone	0.515	0.283–0.917	0.024	0.523	0.277–0.985	0.045
Cardiac tamponade	0.328	0.164–0.626	<0.001	0.434	0.203–0.927	0.031
Cardiac arrest	0.148	0.008–0.849	0.030	0.587	0.056–6.132	0.656
Severe aortic valve regurgitation	0.744	0.263–1.998	0.559			
Coma	0.142	0.022–0.521	0.002	0.364	0.070–1.884	0.228
Neurological deficits of the extremities	0.377	0.178–0.754	0.005	0.369	0.161–0.845	0.018
Hemoglobin (per 0.1 g/dL)	1.012	0.999–1.024	0.065	0.993	0.974–1.011	0.427
Serum creatinine (per 0.1 mg/dL)	0.986	0.961–1.008	0.228			
Serum albumin (per 0.1 g/dL)	1.062	1.011–1.118	0.016	1.030	0.960–1.105	0.414
Total arch replacement	0.701	0.417–1.173	0.177			
Concomitant procedures	0.520	0.263–0.993	0.047	0.504	0.227–1.121	0.093
Operation time (per 10 min)	0.977	0.955–0.999	0.037			
Cardiopulmonary bypass time (per 10 min)	0.955	0.920–0.987	0.006	0.955	0.908–1.002	0.069
Aortic cross-clamp time (per 10 min)	0.944	0.888–0.999	0.048			
Intraoperative fluid balance (per 100 mL)	0.983	0.971–0.995	0.003	0.995	0.980–1.010	0.504

OR, odds ratio; CI, confidence interval; COPD, chronic obstructive pulmonary disease

## Discussion

In this study, we investigated the preoperative and intraoperative predictors of direct home discharge in patients following surgical repair for ATAAD. Multivariable analysis revealed that advanced age, living alone, preoperative cardiac tamponade, and neurological deficits of the extremities were significant independent predictors of a reduced likelihood of direct home discharge.

Our study identified preoperative cardiac tamponade and neurological deficits of the extremities as significant barriers to direct home discharge. Preoperative cardiac tamponade is a well-known predictor of increased in-hospital mortality^10,11)^; however, our findings suggest that survivors often suffer from severe functional decline resulting from systemic malperfusion and the subsequent physical deconditioning associated with preoperative shock. Preoperative neurological deficits of the extremities in ATAAD frequently arise from peripheral limb ischemia, as well as cerebral or spinal malperfusion—the latter of which strongly predisposes patients to postoperative stroke or spinal cord ischemia.^12)^ These neurological insults, particularly lower limb paralysis, directly compromise mobility and functional independence. Similarly, advanced age emerged as a significant negative predictor of direct home discharge. Elderly patients typically possess diminished physiological reserves and require longer postoperative hospital stays,^13)^ indicating that elderly survivors may need more time to recover their functional independence. This relationship is further supported by the significantly lower BI scores at discharge in the non-home discharge group, reflecting a higher dependency in ADLs. Notably, living alone emerged as a significant social factor influencing the discharge destination, independent of clinical severity. For these patients, returning directly home necessitates not only physiological recovery but also the ability to perform all ADLs safely and independently. Consequently, the threshold for home discharge is inherently higher for those living alone compared to patients who benefit from family support.

Interestingly, intraoperative factors—such as concomitant procedures, cardiopulmonary bypass time, and fluid balance—were not identified as independent predictors in our multivariable model. Although cardiopulmonary bypass time, which reflects surgical complexity, is associated with mortality,^14,15)^ our results emphasize that the trajectory of postoperative functional recovery hinges primarily on the patients’ preoperative status: specifically, their physiological resilience, hemodynamic stability, neurological integrity, and social support system. These findings suggest that the primary hurdles to direct home discharge are rooted in baseline vulnerabilities rather than the technical nuances of the procedure itself.

Consequently, our study provides valuable insights for acute care hospitals to facilitate personalized discharge planning and the optimal allocation of rehabilitation resources. Patients characterized by advanced age, living alone, preoperative cardiac tamponade, or neurological deficits of the extremities frequently require post-acute care and intensive rehabilitation. This underscores the fact that multidisciplinary discharge coordination can be initiated based on the preoperative profile. By identifying high-risk individuals at the earliest possible stage, clinicians can establish a proactive foundation for bridging the gap between surgical intervention and functional recovery, regardless of the subsequent postoperative course.

This study has several limitations. First, the single-center, retrospective nature of this study may limit the generalizability of the results, warranting further validation in diverse settings. For instance, a nationwide study reported that two-thirds of patients with ATAAD were discharged directly home,^3)^ whereas only approximately half of our cohort did so. The reasons for this discrepancy remain unclear; however, the influence of unmeasured confounding factors, such as institutional policies, regional differences in healthcare resources, and social security systems, must be considered. Second, this study focused on preoperative and intraoperative factors and did not account for the impact of specific postoperative complications. While postoperative events undoubtedly influence discharge destination, our results highlight the significance of baseline status as an early screening tool. Because our prediction model was designed to rely exclusively on these baseline variables, it consequently demonstrated moderate discriminative ability with an AUC of 0.731. Future studies incorporating postoperative complications will be indispensable to further optimize predictive performance and facilitate more precise decision-making. Third, preoperative functional capacity was not fully characterized in our cohort; patients with pre-event ADL dependency were excluded, and objective measures of frailty were unavailable. Integrating these baseline functional status metrics into future models may further enhance the precision of risk stratification and subsequent discharge coordination.

## Conclusion

Our study demonstrates that advanced age, living alone, preoperative cardiac tamponade, and neurological deficits of the extremities are significant predictors of a reduced likelihood of direct home discharge following ATAAD repair. Recognizing these clinical and social factors at the point of admission enables clinicians to initiate personalized discharge planning, thereby bridging the gap between surgical survival and functional recovery.

## Declarations

### Data availability

The data that support the findings of this study are not publicly available due to privacy restrictions but are available from the corresponding author upon reasonable request.
